# Leucine Supplementation Protects from Insulin Resistance by Regulating Adiposity Levels

**DOI:** 10.1371/journal.pone.0074705

**Published:** 2013-09-25

**Authors:** Elke Binder, Francisco J. Bermúdez-Silva, Caroline André, Melissa Elie, Silvana Y. Romero-Zerbo, Thierry Leste-Lasserre, llaria Belluomo, Adeline Duchampt, Samantha Clark, Agnes Aubert, Marco Mezzullo, Flaminia Fanelli, Uberto Pagotto, Sophie Layé, Gilles Mithieux, Daniela Cota

**Affiliations:** 1 INSERM, Neurocentre Magendie, Physiopathologie de la Plasticité Neuronale, U862, Bordeaux, France; 2 Université de Bordeaux, Neurocentre Magendie, Physiopathologie de la Plasticité Neuronale, U862, Bordeaux, France; 3 IBIMA-Hospital Carlos Haya, Laboratorio de Investigación, Malaga, Spain; 4 INSERM, U855, Lyon, France; 5 Université de Lyon, Lyon, France; 6 Université Lyon 1, Villeurbanne, France; 7 Nutrition et Neurobiologie Intégrée, Université de Bordeaux, UMR 1286, Bordeaux, France; 8 INRA, Nutrition et Neurobiologie Intégrée, UMR 1286, Bordeaux, France; 9 Endocrinology Unit and Centro di Ricerca Biomedica Applicata, Department of Clinical Medicine, S.Orsola-Malpighi Hospital, Alma Mater University of Bologna, Bologna, Italy; State University of Rio de Janeiro, Biomedical Center, Institute of Biology, Brazil

## Abstract

**Background:**

Leucine supplementation might have therapeutic potential in preventing diet-induced obesity and improving insulin sensitivity. However, the underlying mechanisms are at present unclear. Additionally, it is unclear whether leucine supplementation might be equally efficacious once obesity has developed.

**Methodology/Principal Findings:**

Male C57BL/6J mice were fed chow or a high-fat diet (HFD), supplemented or not with leucine for 17 weeks. Another group of HFD-fed mice (HFD-pairfat group) was food restricted in order to reach an adiposity level comparable to that of HFD-Leu mice. Finally, a third group of mice was exposed to HFD for 12 weeks before being chronically supplemented with leucine. Leucine supplementation in HFD-fed mice decreased body weight and fat mass by increasing energy expenditure, fatty acid oxidation and locomotor activity *in vivo*. The decreased adiposity in HFD-Leu mice was associated with increased expression of uncoupling protein 3 (UCP-3) in the brown adipose tissue, better insulin sensitivity, increased intestinal gluconeogenesis and preservation of islets of Langerhans histomorphology and function. HFD-pairfat mice had a comparable improvement in insulin sensitivity, without changes in islets physiology or intestinal gluconeogenesis. Remarkably, both HFD-Leu and HFD-pairfat mice had decreased hepatic lipid content, which likely helped improve insulin sensitivity. In contrast, when leucine was supplemented to already obese animals, no changes in body weight, body composition or glucose metabolism were observed.

**Conclusions/Significance:**

These findings suggest that leucine improves insulin sensitivity in HFD-fed mice by primarily decreasing adiposity, rather than directly acting on peripheral target organs. However, beneficial effects of leucine on intestinal gluconeogenesis and islets of Langerhans's physiology might help prevent type 2 diabetes development. Differently, metabolic benefit of leucine supplementation is lacking in already obese animals, a phenomenon possibly related to the extent of the obesity before starting the supplementation.

## Introduction

Healthy eating is critical for health and well-being. However, despite the evident logic of such a statement, it is a fact that nutrition-related diseases, such as obesity are currently on the rise. Diet composition strongly influences metabolic changes that lead to obesity and its associated diseases, including diabetes, dyslipidemia and cancer [1]. For instance, the consumption of western diets, which are rich in saturated fats and sugars, not only favors obesity, but also plays a significant role in the development of insulin resistance [2]. On the other hand, diets high in proteins and often restricted in carbohydrate intake, are quite popular among overweight and obese subjects pursuing better weight and glycemic control strategies [3], [4]. Thus, manipulation of macronutrient content has an important impact on energy balance regulation and consequently on health, since macronutrients do not only represent fuel substrates but also work as signaling molecules able to affect cellular metabolic processes [5].

Several mechanisms can explain the potential beneficial effects of high-protein diets on the control of body weight. Among those, increased thermogenesis and satiety caused by a higher protein intake are known to facilitate loss of weight and its maintenance afterwards [3], [6]. Branched-chain amino acids (BCAA), including leucine, isoleucine, and valine, are essential amino acids and recent studies support the idea that they may be responsible for some of the beneficial effects of high-protein diets (reviewed in [5]). BCAA are key anabolic signals and, among them, leucine is the most effective in inducing protein synthesis by stimulating the intracellular energy-sensing mammalian target of rapamycin complex 1 (mTORC1) pathway [7], [8]. However, only a series of recent studies carried out in rodents has shown that this amino acid critically affects energy balance regulation [9]-[13]. In particular, several investigations have demonstrated the ability of leucine supplementation to prevent high-fat diet (HFD)-induced obesity and insulin resistance through multiple mechanisms, spanning from increased energy expenditure to decreased hepatic steatosis and reduced inflammation of the adipose tissue [12], [14]. In studies where a clear improvement in insulin sensitivity was described, HFD-fed animals supplemented with leucine were also characterized by decreased adiposity [12], [14], [15]. However, leucine is also known to directly improve insulin signaling in adipocytes [16] and to work as an insulin secretagogue signal in pancreatic β cells [17]. Therefore, it is at present unclear whether the improvement in insulin sensitivity described during the chronic supplementation of leucine to HFD-fed mice is the result of the reduced adiposity or of a direct action of the amino acid onto target tissues. In addition, while supplementation of leucine to HFD-fed animals developing HFD-induced obesity seems overall beneficial, it is uncertain whether same positive effects might be reached by supplementing leucine to already obese animals.

Thus, in the current study we verified whether the improved insulin sensitivity described in animals supplemented with leucine while developing obesity could be explained by their reduced adiposity and also assessed whether the supplementation of the amino acid to already obese mice could beneficially impact body weight, body composition, energy expenditure or glucose homeostasis.

## Materials and Methods

### Ethics Statement

All experiments were conducted in strict compliance with the European Union recommendations (2010/63/EU) and were approved by the French Ministry of Agriculture and Fisheries (animal experimentation authorization n° 3309004) and by the Regional ethical committee of Aquitaine (dossier number: AP1022010). Maximal efforts were made to reduce the suffering and the number of animals used.

### Animals

6-weeks old male C57BL/6J mice (Janvier, Le Genest-Saint-Isle, France) were housed individually in standard plastic rodent cages and maintained at 22°C on a 12-hour light-dark cycle (lights off at 1 P.M.). After 1 week of acclimation, mice were evenly distributed by food intake, body weight and body composition in different treatment groups. **EXP1:** for the study of the effects of leucine on the development of HFD-induced obesity, mice had *ad libitum* access to pelleted chow (3.2 kcal/g, Standard Rodent Diet A03, SAFE, Augy, France) or to a HFD containing 60% calories from fat (5.24 kcal/g, D12492, Research Diets, New Brunswick, NJ, USA) for 17 weeks. Both diets contained 20% protein calories but the regular chow contained 50% of carbohydrate calories, while the HFD comprised 20% of carbohydrate calories. Half of the animals in each diet group received either water or water supplemented with 1.5% (wt/vol) L-leucine (Sigma-Aldrich, St. Louis, MO, USA) since the beginning of the exposure to HFD, as in [12]. **EXP2:** to study the effect of adiposity, a “pairfat” group was generated by slightly restricting for 17 weeks the daily food intake (18% decrease of *ad libitum* food intake) of a group of HFD-fed mice, in order to obtain by the end of the study the same amount of fat, expressed as % of body weight, as in HFD, leucine-supplemented mice. Repeated body composition analysis was carried out to verify comparable fat content between HFD-pairfat and HFD-Leu mice over time. **EXP3:** to assess the effect of leucine supplementation once the animals had become obese, mice were fed HFD *ad libitum* for 12 weeks and subsequently evenly distributed in 2 groups supplemented or not with leucine in drinking water while maintained on HFD. In all studies, food intake and body weight were recorded five times a week. Feed efficiency was calculated over the period of the study as body weight gained per g of food intake. At the end of the study, animals were sacrificed during the light phase by cervical dislocation, tissues collected in ice-cold isopentane and dry ice and stored in −80°C until biochemical analyses were carried out. At time of sacrifice, pancreatic islets were also isolated for subsequent *in vitro* studies. Number of animals used for each experiment is detailed in the figure legends.

### Body composition analysis

A nuclear echo magnetic resonance imaging whole-body composition analyzer (Echo MRI 900; EchoMedical Systems, Houston, TX, USA) was used to repeatedly assess body fat and lean mass in conscious mice.

### Indirect Calorimetry

Mice were individually housed in metabolic chambers (TSE systems GmbH, Bad Homburg, Germany) in which fluid, food intake, in cage locomotor activity and gas exchanges can be monitored. Following 72 hours of acclimation, O2 consumption, CO2 production and locomotor activity were measured continually every 60 minutes for a total of 24 hours to measure the gas exchange, respiratory quotient (RQ), and energy expenditure. Food and water intake were measured continuously by integration of scales into the sealed cage environment. Simultaneously, home-cage locomotor activity was determined using a tri-dimensional infrared light beam system.

### Glucose tolerance test (GTT) and insulin tolerance test (ITT)

Animals were injected with 2 g/kg of D-Glucose (Sigma Aldrich, St Louis, MO, USA) for the GTT or with 0.5 U/kg of insulin (Humulin, Lilly, France) for the ITT. For the GTT, animals were fasted overnight; while for the ITT animals were fasted and leucine-deprived for 7 hours, as in [12]. The tests were conducted the following morning. Blood samples were taken from the tail vein and glucose concentration was measured using glucose sticks (OneTouch Vita, Lifescan France, Issy les Moulineaux, France). At time 0 of the GTT a blood sample was also collected, centrifuged at 3000 rpm for 15 min and the obtained plasma stored at −80°C for subsequent measurement of insulin. HOMA index was calculated using the formula (fasting glucose mmol/L×fasting Insulin mU/L)/22.5.

### Hormonal and lipids analysis

Insulin (plasma levels and *in vitro* secretion) was measured using an ELISA kit from Mercodia (Uppsala, Sweden) following the manufacturer's instructions. Plasma HDL and LDL cholesterol were determined with Abcam ELISA kits (Abcam, Paris, France) and free fatty acids with an Abcam colorimetric assay kit. Hepatic triglycerides were extracted [18] and quantified using a triglyceride colorimetric assay kit (Cayman Chemicals, Ann Arbor, MI, USA).

### Plasma aminoacids and acylcarnitines analysis

Plasma samples were obtained from blood collected from mice in the fed state (4^th^ hour of the dark phase) or in the postabsorptive state (7^th^ hour of the light cycle, after 5 hours food and leucine deprivation) as in [12], after 4 weeks of supplementation with leucine. Plasma amino acids and acylcarnitines levels were measured by liquid chromatography - tandem mass spectrometry (LC-MS/MS) by the AbsoluteIDQ p180 kit by Biocrates Life Sciences AG (Innsbruck, Austria). LC-MS/MS platform was composed by a Serie 200 high pressure liquid chromatograph (HPLC) (Perkin Elmer, Waltham, MA) coupled with a 4000QTrap mass spec (AB-Sciex, Toronto, Canada). Intra-assay coefficient of variation ranged between 4.7 and 15.5% for all analytes

### Quantitative real-time PCR (Q-PCR)

Epididymal white and brown adipose tissues were homogenized in Trizol (Fermentas, Fisher Scientific SAS, Illkirch, France) and RNA was isolated using a standard chloroform/isopropanol protocol. RNA was processed and analyzed as in [19]. Q-PCR reactions were done in duplicate for each sample, using transcript-specific primers, cDNA (4 ng) and LightCycler 480 SYBR Green I Master (Roche) in a final volume of 10 µl. For the determination of the reference gene, the Genorm method was used. Relative expression analysis was corrected for PCR efficiency and normalized against two reference genes (see Table S1). The relative level of expression was calculated using the comparative (2^−*ΔΔ*CT^) method. Primers sequences are reported in Table S1.

### Western blot analysis

Proteins from epididymal white and brown adipose tissues were extracted and quantified and western blots carried out as in [20]. Membranes were incubated with the following primary antibodies: phospho-acetyl-CoA carboxylase (Ser79) (phospho-ACC, 1∶1000, Cell Signaling Technology, Beverly, MA), acetyl-CoA carboxylase 1 (1∶1000, Cell Signaling), fatty acid synthase (FAS, 1∶1000, Cell Signaling), anti-uncoupling protein 3 (UCP-3, 1∶1000 Sigma) and β-Actin (1∶1000, Cell Signaling), which was used as loading control. Immunoreactive bands were visualized using enhanced chemiluminescence (ECL plus, PerkinElmer) then exposed on radiographic films. Bands quantification was performed using Image J software (National Institute of Health, Bethesda, Maryland, USA).

### Islets isolation and in vitro glucose-stimulated insulin secretion

All reagents used were from Sigma-Aldrich. Pancreatic islets were isolated by collagenase digestion method [21]. Briefly, pancreas was inflated with Hanks solution containing 0.8 mg/ml of collagenase, 5.6 mM glucose and 0.05% bovine serum albumin, pH 7.4, then the tissue was removed and kept at 37°C for 6–8 min. After tissue digestion, the islets were manually collected and left recovering in RPMI culture media for 18–20 hours. For static incubation experiments, groups of size-matched five islets were first incubated for 1 h at 37°C in 0.5 ml Krebs-bicarbonate buffer solution (in mM): 115 NaCl, 5 KCl, 2.56 CaCl2, 1 MgCl2, 24 NaHCO3, 15 HEPES and 5.6 glucose, supplemented with 0.05% of bovine serum albumin and equilibrated with a mixture of 95% O2: 5% CO2, pH 7.4. The medium was then replaced with 0.5 ml fresh buffer containing 3 mM glucose or 11 mM glucose and further incubated for 1 h. The islets were subsequently put at 4°C for 15 min to stop insulin secretion and the media were collected, centrifuged at 1200 rpm for 10 min at 4°C and stored at −20°C for measurement of insulin content by ELISA (Mercodia).

### Immunohistochemistry

Pancreases from water- or leucine-supplemented HFD-fed mice were fixed in 4% paraformaldehyde in PBS by immersion, dehydrated and embedded in paraffin. Tissue blocks were cut into 5 µm-thick sections using a Microm HM325 microtome (MICROM, Walldorf, Germany). Sections were dewaxed, washed several times with PBS, and incubated in 3% hydrogen peroxide in PBS for 10 min in the dark at room temperature in order to inactivate endogenous peroxidase. After 3 washes in PBS for 5 min, sections were incubated in blocking solution containing 10% goat serum, 0.2% triton X-100, and 0.1% sodium azide for 1 h, followed by overnight incubation at room temperature with mouse anti-insulin antibody (diluted 1∶200; Sigma). Sections were then washed three times with PBS, incubated in a biotinylated goat anti-mouse IgG (diluted 1∶500; Sigma) for 1 h, washed again in PBS, and incubated in ExtrAvidin peroxidase (diluted 1∶2000; Sigma) for 1 h. After three washes in PBS, immunolabeling was revealed with 0.05% diaminobenzidine (DAB; Sigma) and 0.03% H_2_O_2_ in PBS. Sections were counterstained with haematoxilin and then dehydrated in ethanol, cleared in xylene, and coverslipped with Eukitt mounting medium (Kindler GmBH and Co., Freiburg, Germany).

Digital high-resolution microphotographs were taken under the same conditions of light and brightness/contrast using an Olympus BX41 microscope equipped with a 10× objective and an Olympus DP70 digital camera (Olympus Europa GmbH, Hamburg, Germany). Quantification of signal was carried out by densitometric analysis carried out on two images per sample, five samples per group, using software Image J. Additionally, islet numbers were counted on whole pancreatic sections and islet surface was quantified by marking islet borders using Image J.

#### Intestinal Gluconeogenesis

The proximal jejunum was rinsed in ice-cold 0.1 M PBS and immediately frozen. Glucose-6-phosphatase (G6Pase) and phosphoenolpyruvate carboxykinase (PEPCK) activities were assayed as described previously [22].

### Statistics

All values are reported as mean±SEM. Statistics were performed using Statistica version 9 (Statsoft, Maisons-Alfort, France). Body weight, energy expenditure, GTT and ITT glucose curves were analyzed with repeated measures ANOVA, and Tukey's HSD post-hoc test was applied when appropriated. *In vitro* insulin secretion, feed efficiency, respiratory quotient and locomotor activity, were analyzed with 2-way ANOVA, while body composition, G6Pase activity, islets number and insulin content, hormonal, lipid and amino acid measurements, Q-PCR and western blot data were analyzed with a two-tailed t-test. *p* values less than 0.05 denote statistical significance.

## Results

### Leucine supplementation protects from body weight gain during HFD exposure by increasing energy expenditure and fatty acid oxidation *in vivo*

**Figure 1:**
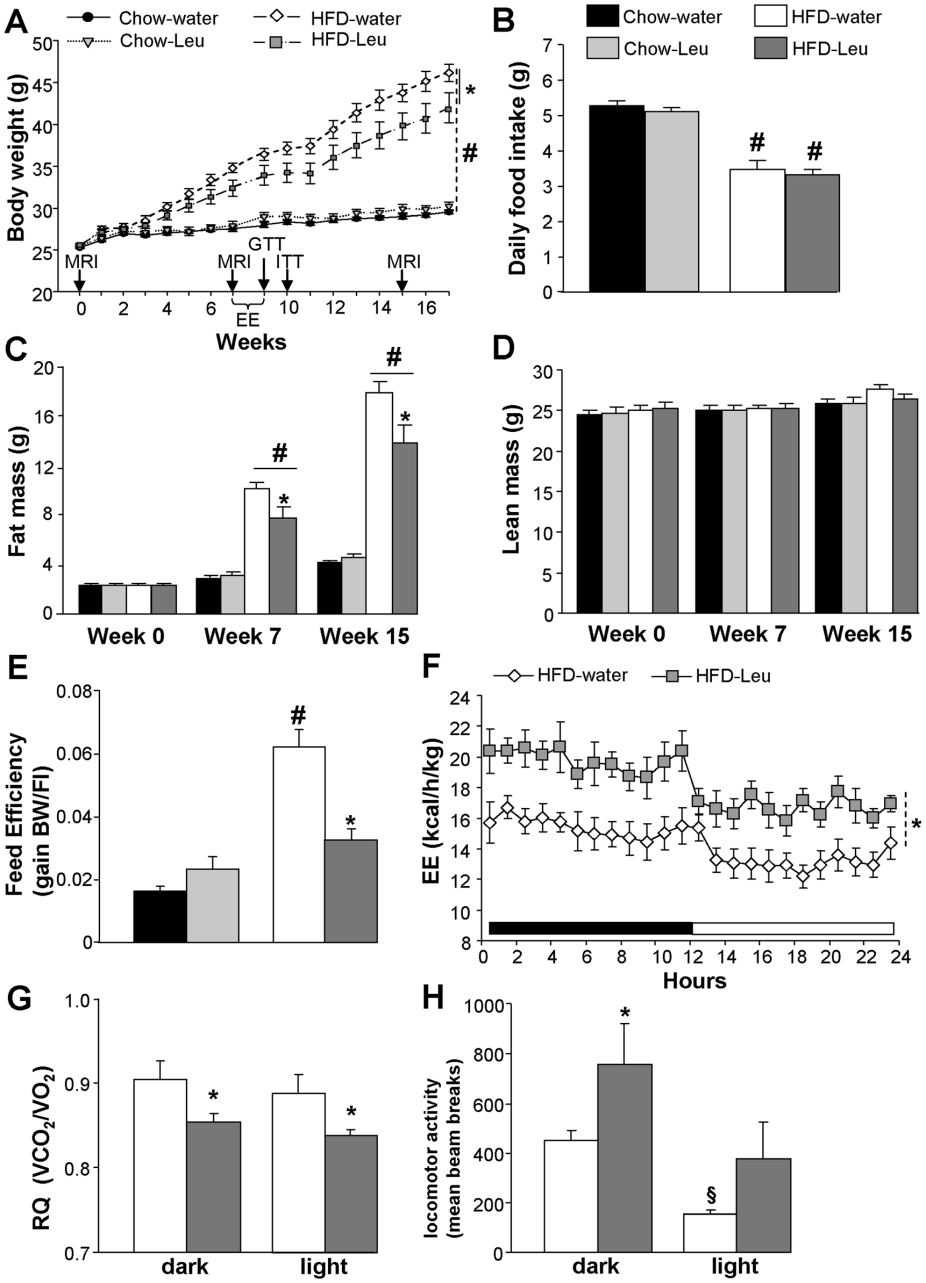
Leucine supplementation protects from HF-induced body weight gain and adiposity by increasing energy expenditure and fatty acid oxidation. (A) Body weight curve and (B) mean daily food intake of mice exposed to HFD or chow and supplemented or not with leucine in drinking water (n = 10–11 per group). (C) Fat mass and (D) lean mass at week 0 (baseline), as well as at weeks 7 and 15 of the study (n = 10–11 per group). (E) Feed efficiency in chow-fed and HFD-fed mice supplemented or not with leucine in drinking water (n = 10–11 per group). Energy expenditure (F), respiratory quotient (G) and locomotor activity (H) in HFD-fed mice supplemented or not with leucine in drinking water (n = 6–8 per group). Arrows in (A) indicates when *in vivo* experiments were carried out. EE: energy expenditure analysis; MRI: magnetic resonance imaging whole-body composition analysis; RQ: respiratory quotient. ^*^ *p*<0.05 vs. HFD-water group; ^#^ *p*<0.05 vs. chow-fed groups, ^§^ *p*<0.05 vs. dark phase.

The mice on chow and on HFD respectively ingested 74.5±1.2 mg/day and 55.6±2.4 mg/day of leucine from the diet. Leucine-supplemented mice in each diet group ingested an additional 97.9±6.2 mg (chow-group) and 52.2±4.7 mg (HFD-group) daily via water consumption, roughly doubling their total daily leucine intake. HFD-Leu mice gained significantly less weight as compared to water, HFD-fed control animals (Figure 1A). Conversely, leucine supplementation did not affect body weight (Figure 1A) or body composition (Figure 1, C and D) in chow-fed animals. The decrease in body weight gain observed in HFD-Leu mice was due to a decrease in fat mass (Figure 1C), while no changes were observed in lean mass (Figure 1D) or food intake as compared to HFD-water animals (Figure 1B). HFD-fed mice ingested significantly less food than chow-fed animals (Figure 1B). However when expressed in Kcal/day, the mice had comparable caloric intake (chow-water: 15.89±0.3 kcal/day; chow-Leu: 15.42±0.4 kcal/day; HFD-water: 16.67±0.8 kcal/day; HFD-Leu: 15.75±0.8 kcal/day, *p*>0.05). Consequently, and as compared to the HFD-water group, a significant decrease in feed efficiency became evident in HFD-Leu mice (Figure 1E, *p*<0.05), which was associated to a significant increase in energy expenditure, present during both the dark and light phase of the diurnal cycle (Figure 1F). In addition, HFD-Leu mice showed a significant decrease in the respiratory quotient (Figure 1G), suggestive of increased *in vivo* fatty acid oxidation, which was associated to increased locomotor activity during the dark phase (Figure 1H). The latter might in turn have contributed to the observed increase in energy expenditure.

**Figure 2:**
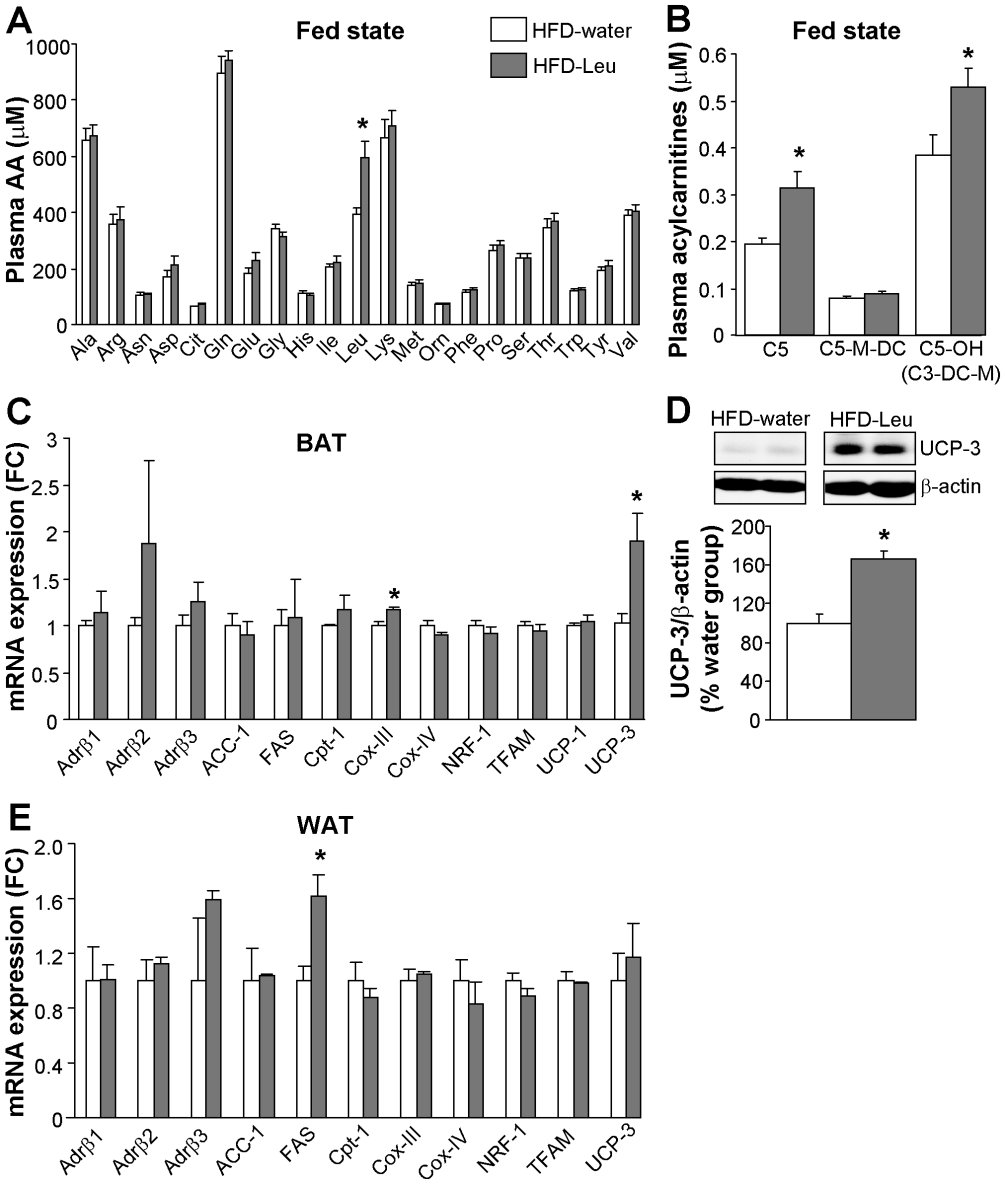
Plasma leucine levels and molecular changes induced by leucine supplementation in HFD-fed mice. (**A**) Plasma amino acids and (**B**) acylcarnitines deriving from leucine metabolism measured during the fed state in HFD-fed mice supplemented or not with leucine in drinking water (n = 9 per group). (**C** and **E**) mRNA expression levels of several markers of mitochondrial activity, fatty-acid metabolism and adrenergic β-receptor subtypes in the BAT (C) and WAT (E) of HFD-fed mice supplemented or not with leucine in drinking water (n = 5 per group). (**D**) Protein expression of UCP-3 in the BAT of HFD-fed mice supplemented or not with leucine in drinking water (n = 5 per group), β-actin loading control. C5: isovalerylcarnitine; C5-OH (C3-DC-M): hydroxyl-isovalerylcarnitine; C5-MDC: methylglutarylcarnitine; FC: fold change.^ *^ *p*<0.05 vs. HFD-water group.

When plasma amino acids were assessed in HFD-fed mice, we found a significant increase only in plasma leucine in animals supplemented with leucine (HFD-Leu group; Figure 2A), which was associated to increased levels of plasma acylcarnitines deriving from leucine metabolism (Figure 2B). These increases were present in the fed state, but not in the postabsorptive state (data not shown). Subsequent *ex-vivo* molecular analysis of the brown adipose tissue (BAT) from HFD-Leu and HFD-water mice showed a significant increase in the mRNA expression levels of the mitochondrial markers cyclooxygenase-III (Cox-III) and uncoupling protein 3 (UCP-3) in the HFD-Leu group (Figure 2C). UCP-3 protein expression was also increased in the BAT (Figure 2D). Differently, in the epididymal white adipose tissue (WAT), apart from an increase in the mRNA expression of the fatty acid synthase (FAS) in the HFD-Leu group (Figure 2E), no significant changes were observed, neither in the expression of genes of mitochondrial activity and lipid metabolism, nor of the β-adrenergic receptor subtypes (Figure 2E). Additionally in the WAT, FAS protein levels and acetyl-CoA carboxylase (ACC) phosphorylation at Ser79, which is regulated by AMP-activated protein kinase (AMPK) activity [23], were comparable between HFD-Leu and HFD-water mice (data not shown). Finally, HFD-water and HFD-Leu mice had similar plasma free fatty acids, HDL and LDL cholesterol levels (Table 1).

**Table 1:** Plasma lipid levels in HFD-water and HFD-Leu mice (N = 6). Data reported are expressed as mean±SEM.

	HFD-water	HFD-Leu	*P*
Plasma free fatty acid (nmol/mL)	329.1±34	401.5±86	N.S.
Plasma HDL cholesterol (mmol/L)	12.8±0.6	13.0±0.6	N.S.
Plasma LDL cholesterol (mmol/L)	11.8±0.9	13.2±0.2	N.S.

### Leucine supplementation improves insulin sensitivity

**Figure 3:**
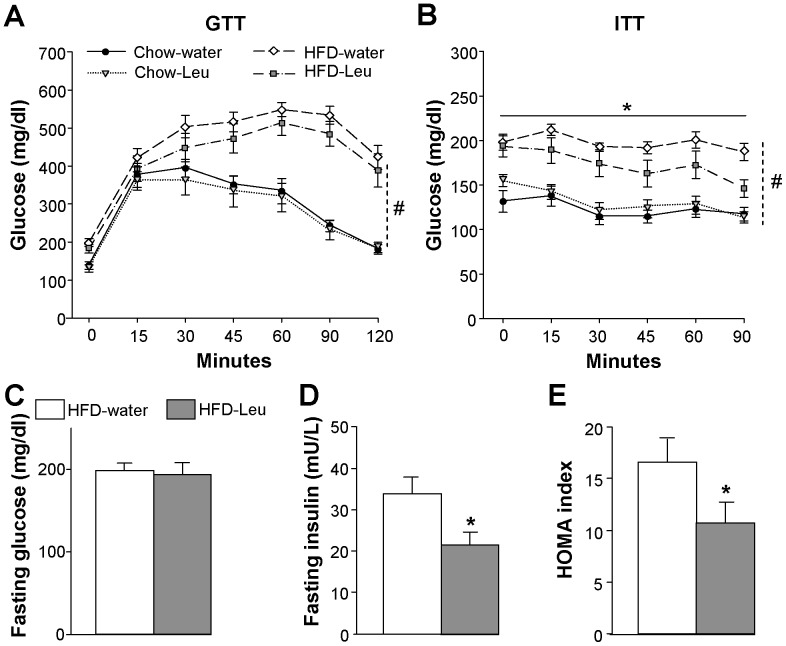
Leucine supplementation in HF-fed mice improves insulin sensitivity. (**A**) Glucose tolerance test and (**B**) insulin tolerance test carried out in HFD- or chow-fed mice supplemented or not with leucine in drinking water (n = 10-11 per group). (**C**) Fasting plasma glucose, (**D**) fasting plasma insulin and (**E**) HOMA index measured in mice on HFD supplemented or not with leucine in drinking water (n = 10-11 per group). ^*^ *p*<0.05 vs. HFD-water group; ^#^ *p*<0.05 vs. chow-fed groups.

Under chow, leucine supplementation did not have any effect on glucose responses, neither during a GTT nor an ITT (Figure 3, A and B). As expected, chow-fed animals had better GTT and ITT curves than HFD-fed mice, with significantly lower levels of plasma glucose over the length of the tests (Figure 3, A and B). No significant differences between HFD-Leu and HFD-water mice were observed in the GTT, although a trend towards decreased glucose levels was observed in the HFD-Leu group (Figure 3A). In the ITT, HFD-Leu animals had significantly lower plasma glucose levels after the administration of insulin, thus showing greater insulin sensitivity than HFD-water controls (Figure 3B). In addition, although fasting glucose levels were comparable between HFD-Leu and HFD-water groups (Figure 3C), fasting plasma insulin levels were significantly lower in HFD-Leu mice as compared to the HFD-water group (Figure 3D), leading to a decreased HOMA index in HFD-fed, leucine-supplemented mice (Figure 3E).

**Figure 4:**
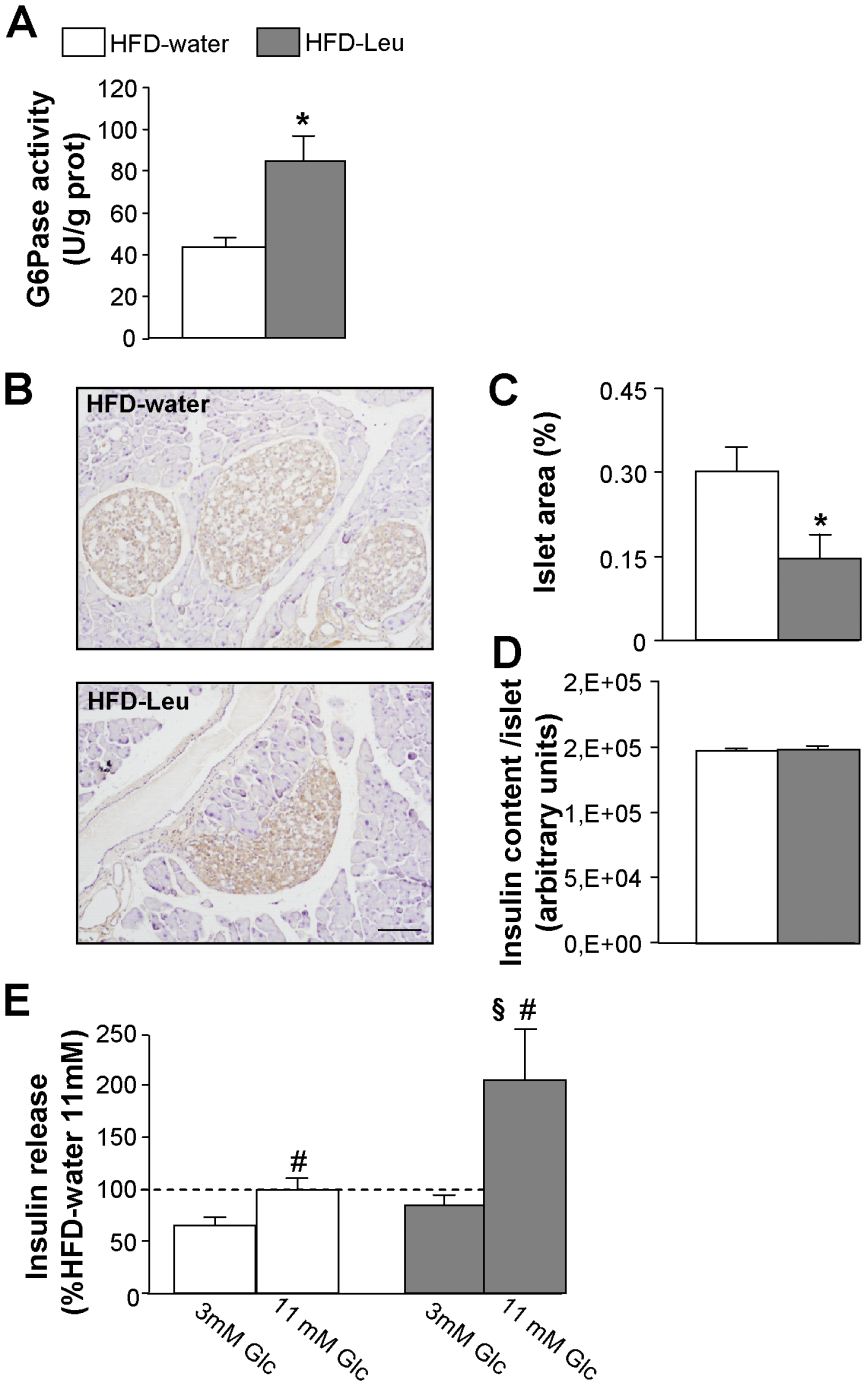
Leucine supplementation in HF-fed mice increases intestinal gluconeogenesis and glucose-stimulated insulin release. (A) G6Pase activity in the jejunum of HFD-fed mice supplemented or not with leucine in drinking water (n = 6 per group). (B) Representative images of Langerhans islets (in brown: insulin staining), (C) islet area and (D) islet insulin content from HFD-fed mice supplemented or not with leucine in drinking water (n = 5 per group). (E) Glucose-stimulated insulin secretion from Langerhans islets of HFD-fed mice supplemented or not with leucine (n = 3 mice per group, two independent experiments). Data are relative to 11 mM secretion in HFD-water mice that was considered 100%. ^*^ *p*<0.05 vs. HFD-water group; ^#^ *p*<0.05 vs. 3 mM condition in each group; ^§^ *p*<0.05 vs. all other groups. Scale bar in (B): 100 µm.

Intestinal gluconeogenesis is among the mechanisms known to affect insulin sensitivity in response to protein ingestion [24]. Interestingly, we found that HFD-Leu mice were characterized by a significant increase in the intestinal activity of the G6Pase, the enzyme regulating the final step of gluconeogenesis [25] (Figure 4A), while levels of PEPCK in this tissue were comparable between HFD-Leu and HFD-water mice (data not shown). In addition, histological analysis of pancreatic islets of Langerhans showed that, while islet insulin content was comparable (Figure 4D), islets area was significantly decreased in HFD-Leu mice as compared to HFD-water animals (Figure 4, B and C), implying that the chronic supplementation of leucine protected from the development of the hypertrophy usually observed in islets from obese, insulin-resistant animals [26]. Moreover, islets from HFD-water animals showed vacuolization and structural disorganization, histopathological hallmarks of diabetes that were not present in HFD-Leu islets. Lastly, *in vitro* studies evidenced a greater glucose-stimulated insulin secretion in islets from animals chronically supplemented with leucine as compared to HFD-water controls (Figure 4E), implying an improved functionality of the islets under leucine supplementation.

### Reduced adiposity suffices to improve insulin sensitivity

**Figure 5:**
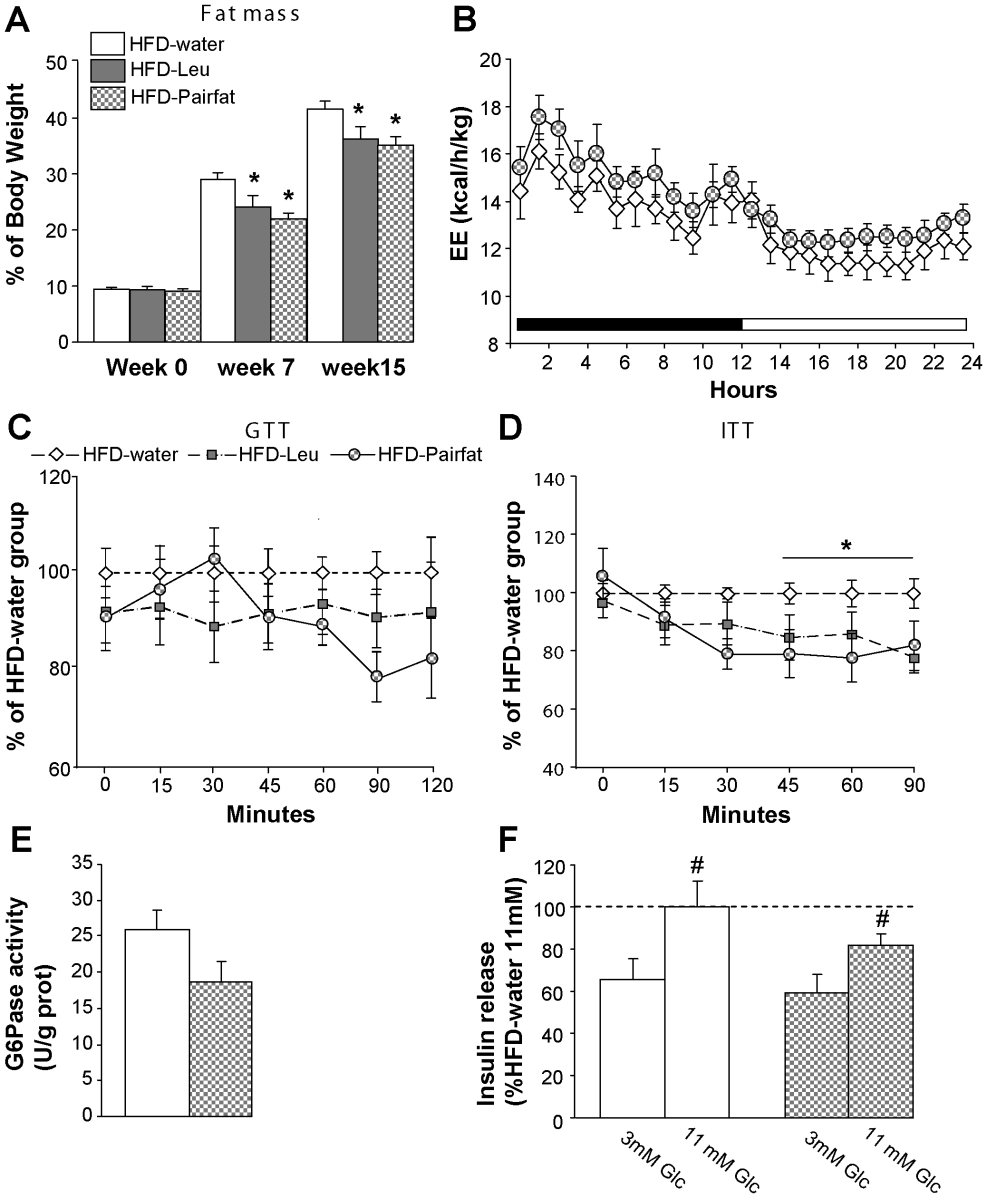
Reduced adiposity is sufficient to improve insulin sensitivity. (**A**) Fat mass, expressed as % of body weight, in HFD-fed mice supplemented or not with leucine and in HFD-pairfat mice (n = 10–11 per group). (**B**) Energy expenditure in HFD-fed water and pairfat mice (n = 6 per group). (**C**) Glucose tolerance and (**D**) insulin tolerance tests carried out in HFD-fed mice supplemented or not with leucine and in pairfat mice. Data are expressed as % of the HFD-water group (n = 10–11 per group). (**E**) G6Pase activity measured in the jejunum of HFD-fed water and pairfat mice (n = 6 per group). (**F**) Glucose-stimulated insulin secretion from Langerhans islets of HFD-fed water and pairfat mice (n = 3 mice per group, two independent experiments). Data are relative to 11 mM secretion in HFD-water mice that was considered 100%. ^*^ *p*<0.05 vs. HFD-water group; ^#^ *p*<0.05 vs. 3 mM condition in each group.

Having observed improved insulin sensitivity in HFD mice supplemented with leucine we wondered whether such improvement was the result of the reduced adiposity that characterizes HFD-Leu mice or rather depended on the above-described action of the amino acid on target organs, such as the gastro-intestinal tract or the endocrine pancreas. Therefore, we generated a group of HFD-fed mice, which was slightly and chronically food restricted over time in order to reach a fat mass comparable to the one of HFD-Leu mice (see Methods, HFD-pairfat group and Figure 5A). HFD-pairfat animals had energy expenditure similar to HFD-water mice (Figure 5B). In addition, HFD-pairfat mice responded comparably to HFD-Leu mice when undergoing a GTT (Figure 5C), while showing the same degree of improvement in insulin sensitivity as HFD-Leu mice during an ITT (Figure 5D). However, and differently from HFD-Leu mice, HFD-pairfat animals did not show any changes in gastro-intestinal G6Pase activity (Figure 5E) or in glucose-stimulated insulin secretion (Figure 5F) when compared to HFD-water mice. Importantly, both HFD-Leu and HFD-pairfat mice had a significant decrease in hepatic triglycerides content when compared to HFD-water controls (hepatic TG content HFD-water: 12.51±1.34 mg/g, HFD-Leu: 6.35±1.23 mg/g, HFD-pairfat: 4.98±0.52 mg/g; *p*<0.05 vs. HFD-water).

### Leucine supplementation in already obese animals does not improve the obese phenotype

**Figure 6:**
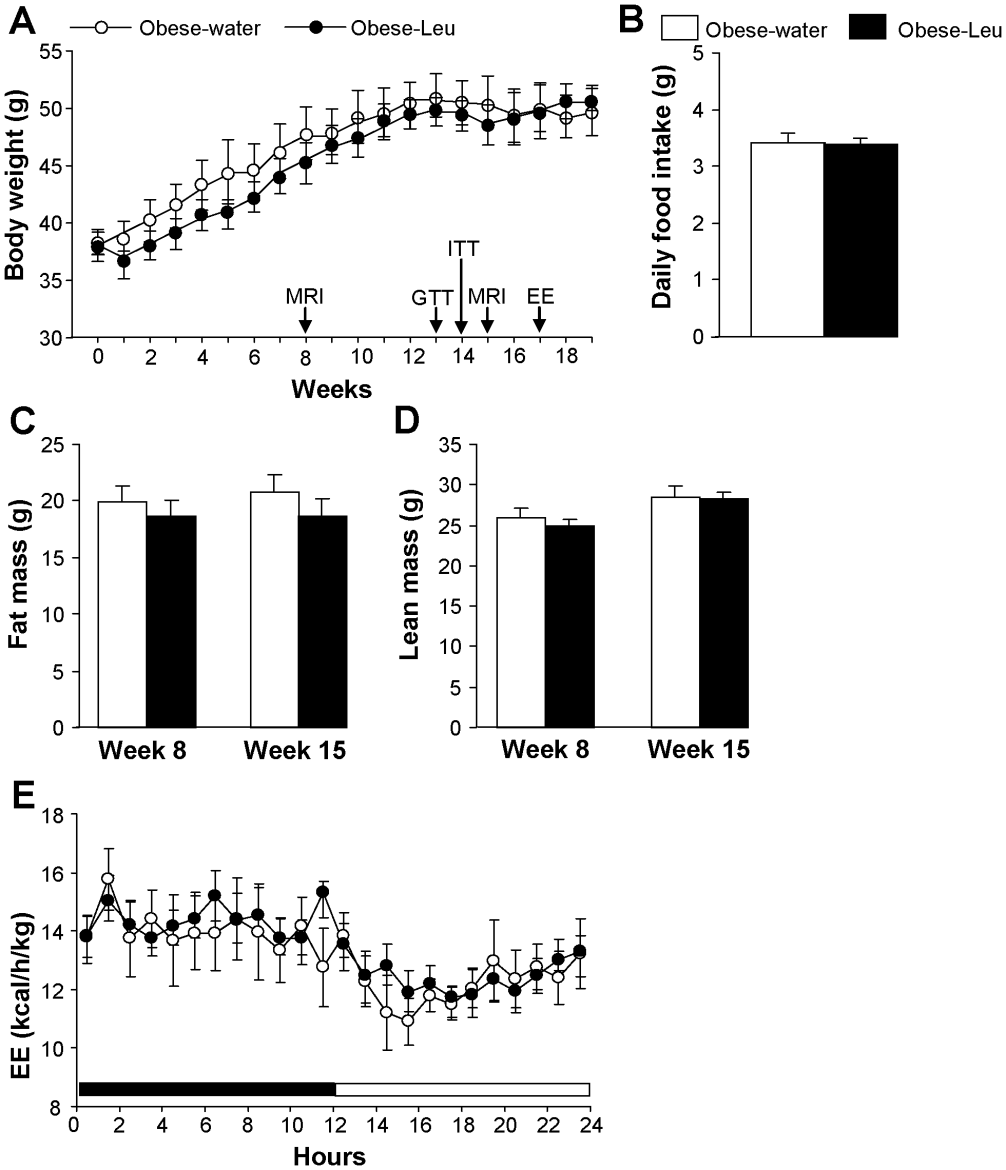
Leucine supplementation in already obese mice does not affect body weight, adiposity or energy expenditure. (**A**) Body weight curve, (**B**) mean daily food intake, (**C**) fat mass and (**D**) lean mass composition and (**E**) 24-h energy expenditure in obese mice supplemented or not with leucine in drinking water while maintained HFD (n = 6–7 mice per group). Arrows in (**A**) indicates when *in vivo* experiments were carried out. EE: energy expenditure analysis; MRI: magnetic resonance imaging whole-body composition analysis.

**Figure 7:**
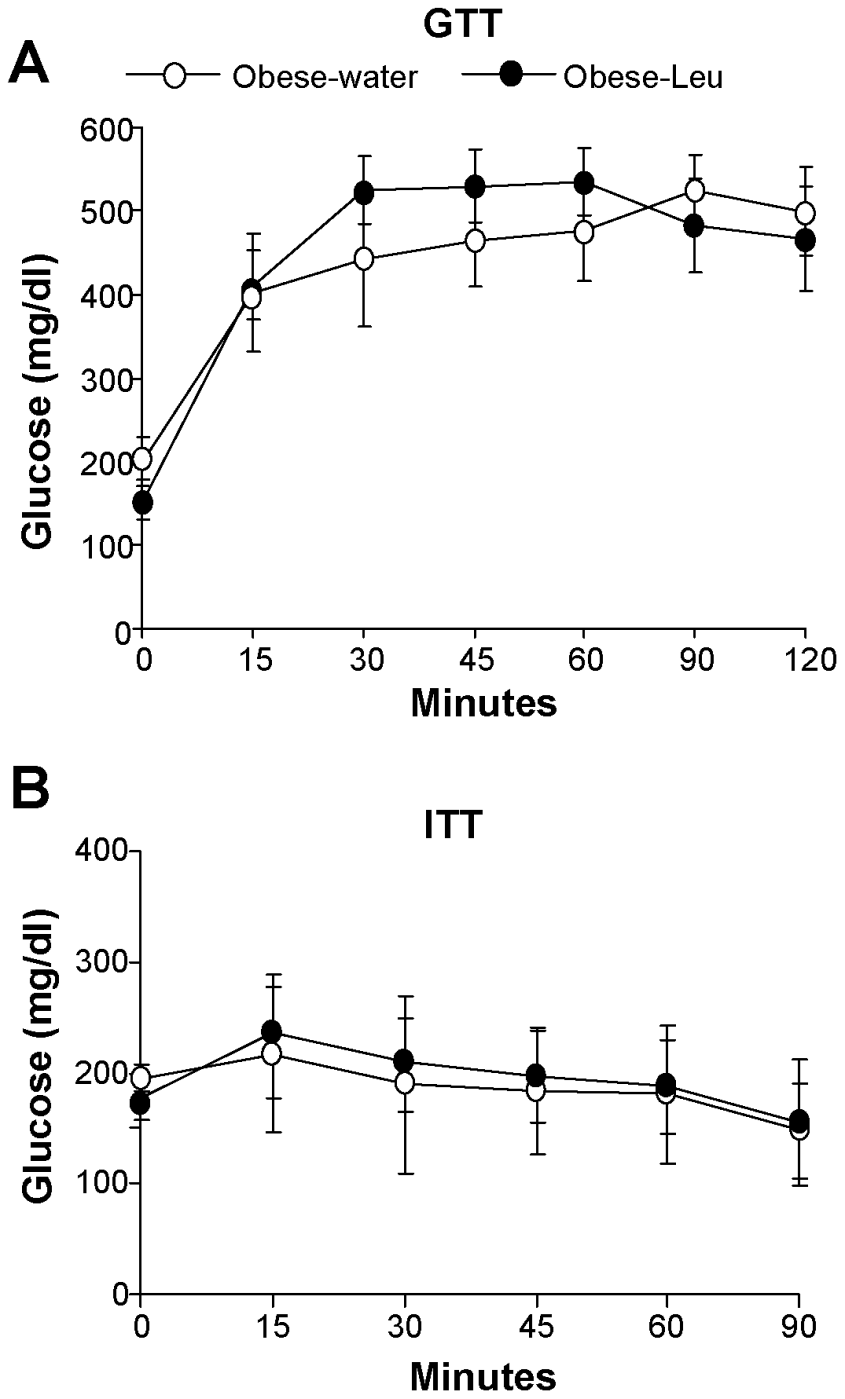
Leucine supplementation in already obese animals does not affect glucose homeostasis. (**A**) Glucose tolerance and (**B**) insulin tolerance tests carried out in obese mice respectively after 13 weeks and 14 weeks of supplementation or not with leucine in drinking water (n = 7per group).

We finally tested whether leucine supplementation through drinking water might improve the metabolic phenotype of mice that have already developed obesity. To this scope mice were fed a HFD for 12 weeks and thereafter supplemented with leucine while continuing to consume the HFD. Under this condition, leucine supplementation did not significantly affect body weight, food intake or body composition (Figure 6 A-D). In addition, no differences in energy expenditure were observed between obese mice supplemented or not with leucine (Figure 6E). Lastly, no improvement in glucose responses was observed in obese mice supplemented with leucine, neither during the GTT or the ITT (Figure 7, A and B).

## Discussion

The present study demonstrates that dietary leucine prevents HFD-induced obesity by increasing energy expenditure, locomotor activity and fatty acid oxidation *in vivo,* consequently leading to decreased adiposity and better insulin sensitivity.

The decreased body weight and fat mass observed in HFD-Leu mice are in agreement with recently published studies describing these effects [12], [14], [15]. It is important to highlight that changes in adiposity were reported in the literature even without apparent changes in body weight [14]. In addition, we were also able to replicate previous evidence showing that chronic supplementation of leucine increases energy expenditure in HFD-fed mice [12], together with an increase in the oxidation of lipid substrates that might have accounted for the observed decrease in fat mass. Molecular analysis also showed that UCP-3 protein expression, an uncoupling protein involved in the regulation of fatty acid oxidation [27], was significantly induced in the BAT. However, leucine supplementation did not induce other relevant changes in lipid metabolism, since circulating free fatty acids, cholesterol fractions and enzymes regulating fatty acid synthesis and oxidation were similar in BAT and WAT, possibly because *ex-vivo* studies were carried out several weeks after the *in vivo* determination of energy expenditure and fatty acid oxidation, when potential differences between HFD-water and HFD-Leu mice were not anymore evident.

As expected, circulating levels of leucine were significantly increased during the fed state (when water consumption is increased due to food intake) in HFD leucine-supplemented mice. Accordingly, we also found increased plasma acylcarnitines deriving from leucine metabolism, implying that leucine was being oxidized at cellular level. Interestingly, *in vitro* studies have recently shown that leucine increases oxidative metabolism and capacity in different muscle cell models [28], while other *in vitro* investigations carried out on LT3-L1 adipocytes have shown that leucine increases fatty acid oxidation, as determined by palmitate oxidation, possibly by increasing the activity of the NAD-dependent deacetylase sirtuin-1 (SIRT-1), whose activation increases mitochondrial function [29]. Thus, the present findings together with the published literature suggest that leucine might affect cellular metabolism by critically regulating the use of fuel substrates, oxidative metabolism and mitochondrial activity.

In all the studies in which leucine supplementation caused a decrease in fat mass, the authors also described a clear improvement in glucose metabolism [12], [14], [15]. Interestingly, this beneficial effect seems related to leucine supplementation only, since when HFD-fed animals were supplemented with a BCAA mix, they developed insulin resistance [30]. In agreement with the published evidence, we also found that HFD mice supplemented with leucine were characterized by improved insulin sensitivity, as suggested by their HOMA index and their glucose responses to an ITT. In our study, leucine supplementation also increased intestinal gluconeogenesis and glucose-stimulated insulin secretion, phenomena that might have both a positive impact on glucose homeostasis. These metabolic improvements were not present in HFD-pairfat animals and as such they did not have direct effects on insulin sensitivity in our model, although they might help delay the development of overt diabetes by participating, together with the decreased adiposity, to lower β cells lipotoxicity and therefore preserve islets physiology.

Notably, the use of the pairfat model in the current study allows concluding that the improvement of insulin sensitivity induced *in vivo* by leucine supplementation first and foremost depends on the ability of the amino acid to impede fat mass accumulation, rather than on the modulation of the function of tissues known to participate to the regulation of glucose homeostasis. As a consequence of the decreased fat accumulation in the organism, both HFD-Leu and HFD-pairfat animals had decreased triglycerides content in the liver, a finding that is in agreement with other published observations describing decreased hepatic lipid accumulation and expression of lipogenic enzymes in the liver of HFD mice supplemented with leucine [14]. Hepatic lipid deposition and consequent activation of inflammatory responses are known to play a critical role in the development of insulin resistance [2], [31]. Even though we did not assess inflammatory markers in liver or adipose tissue, it is likely that both HFD-Leu and HFD-pairfat mice were characterized by decreased inflammation and that these changes might ultimately have led to the improved insulin sensitivity.

A latter aspect that we investigated was the ability of leucine supplementation to affect body weight, adiposity or glucose metabolism in mice that had already developed obesity. This point is rather unexplored, since up to now most of the literature in the field has focused on the action of leucine supplementation during the development of obesity. Our study shows that the amino acid supplementation did not significantly affect any of the studied parameters in already obese mice. A possibility for this lack of effect might be due to a metabolic point of “no return” reached by the animals kept for 12 weeks on the HFD before the supplementation with leucine. Indeed, in a recently published study leucine supplementation was able to decrease adiposity and improve insulin sensitivity in rats that were exposed to a high-fat/high-sucrose diet for only 6 weeks before being switched to the same diet enriched with the amino acid [32]. Nevertheless, the negative evidence described here deserves attention, since it suggests that possible nutritional interventions with leucine supplementation in obese humans might not be helpful.

In conclusion, the present findings strengthen current evidence suggesting a beneficial role for leucine in the prevention of diet-induced obesity and pinpoint its ability to modulate adiposity levels as critical in determining a better metabolic profile. Moreover, our study also provides novel evidence on peripheral leucine actions that might help halt the development of type 2 diabetes. Data collected so far also lead to suggest that leucine supplementation in already obese animals may or may not be able to beneficially improve metabolic parameters depending on the length of the exposure to an obesogenic diet.

Interestingly, a very recent epidemiological study has demonstrated that increased dietary intake of BCAA in humans is inversely associated with prevalence of overweight and obesity in western countries, like UK and US, thus suggesting that increased intake of BCAA might help prevent body weight gain [33]. This evidence, together with our results and a growing amount of experimental animal literature provides a rationale to further explore the role of leucine supplementation in the prevention of weight gain and type 2 diabetes in humans.

## Supporting Information

Table S1 Mouse qPCR primer sequences.
(DOC)

## References

[1] Popkin BM (2007) Understanding global nutrition dynamics as a step towards controlling cancer incidence. Nat Rev Cancer 7: 61–67. 17186019 10.1038/nrc2029

[2] Wisse BE, Kim F, Schwartz MW (2007) Physiology. An integrative view of obesity. Science 318: 928–929. 17991852 10.1126/science.1148032

[3] Westerterp-Plantenga MS, Lemmens SG, Westerterp KR (2012) Dietary protein - its role in satiety, energetics, weight loss and health. Br J Nutr 108 Suppl 2S105–112. 23107521 10.1017/S0007114512002589

[4] Larsen TM, Dalskov SM, van Baak M, Jebb SA, Papadaki A, et al (2010) Diets with high or low protein content and glycemic index for weight-loss maintenance. N Engl J Med 363: 2102–2113. 21105792 10.1056/NEJMoa1007137 PMC3359496

[5] Andre C, Cota D (2012) Coupling nutrient sensing to metabolic homoeostasis: the role of the mammalian target of rapamycin complex 1 pathway. Proc Nutr Soc 71: 502–510. 22877732 10.1017/S0029665112000754

[6] Halton TL, Hu FB (2004) The effects of high protein diets on thermogenesis, satiety and weight loss: a critical review. J Am Coll Nutr 23: 373–385. 15466943 10.1080/07315724.2004.10719381

[7] Proud CG (2007) Amino acids and mTOR signalling in anabolic function. Biochem Soc Trans 35: 1187–1190. 17956308 10.1042/BST0351187

[8] Laplante M, Sabatini DM (2012) mTOR signaling in growth control and disease. Cell 149: 274–293. 22500797 10.1016/j.cell.2012.03.017 PMC3331679

[9] Cota D, Proulx K, Smith KA, Kozma SC, Thomas G, et al (2006) Hypothalamic mTOR signaling regulates food intake. Science 312: 927–930. 16690869 10.1126/science.1124147

[10] Blouet C, Jo YH, Li X, Schwartz GJ (2009) Mediobasal hypothalamic leucine sensing regulates food intake through activation of a hypothalamus-brainstem circuit. J Neurosci 29: 8302–8311. 19571121 10.1523/JNEUROSCI.1668-09.2009 PMC2740923

[11] Blouet C, Schwartz GJ (2012) Brainstem nutrient sensing in the nucleus of the solitary tract inhibits feeding. Cell Metab 16: 579–587. 23123165 10.1016/j.cmet.2012.10.003 PMC3537851

[12] Zhang Y, Guo K, LeBlanc RE, Loh D, Schwartz GJ, et al (2007) Increasing dietary leucine intake reduces diet-induced obesity and improves glucose and cholesterol metabolism in mice via multimechanisms. Diabetes 56: 1647–1654. 17360978 10.2337/db07-0123

[13] Ropelle ER, Pauli JR, Fernandes MF, Rocco SA, Marin RM, et al (2008) A central role for neuronal AMP-activated protein kinase (AMPK) and mammalian target of rapamycin (mTOR) in high-protein diet-induced weight loss. Diabetes 57: 594–605. 18057094 10.2337/db07-0573

[14] Macotela Y, Emanuelli B, Bang AM, Espinoza DO, Boucher J, et al (2011) Dietary leucine--an environmental modifier of insulin resistance acting on multiple levels of metabolism. PLoS One 6: e21187. 21731668 10.1371/journal.pone.0021187 PMC3120846

[15] Li H, Xu M, Lee J, He C, Xie Z (2012) Leucine supplementation increases SIRT1 expression and prevents mitochondrial dysfunction and metabolic disorders in high-fat diet-induced obese mice. Am J Physiol Endocrinol Metab 303: E1234–1244. 22967499 10.1152/ajpendo.00198.2012 PMC3517633

[16] Hinault C, Mothe-Satney I, Gautier N, Lawrence JC Jr, Van Obberghen E (2004) Amino acids and leucine allow insulin activation of the PKB/mTOR pathway in normal adipocytes treated with wortmannin and in adipocytes from db/db mice. FASEB J 18: 1894–1896. 15479767 10.1096/fj.03-1409fje

[17] Sener A, Malaisse WJ (1980) L-leucine and a nonmetabolized analogue activate pancreatic islet glutamate dehydrogenase. Nature 288: 187–189. 7001252 10.1038/288187a0

[18] Pitman JL, Bonnet DJ, Curtiss LK, Gekakis N (2011) Reduced cholesterol and triglycerides in mice with a mutation in Mia2, a liver protein that localizes to ER exit sites. J Lipid Res 52: 1775–1786. 21807889 10.1194/jlr.M017277 PMC3173003

[19] Cardinal P, Bellocchio L, Clark S, Cannich A, Klugmann M, et al (2012) Hypothalamic CB1 cannabinoid receptors regulate energy balance in mice. Endocrinology 153: 4136–4143. 22778221 10.1210/en.2012-1405

[20] Proulx K, Cota D, Woods SC, Seeley RJ (2008) Fatty acid synthase inhibitors modulate energy balance via mammalian target of rapamycin complex 1 signaling in the central nervous system. Diabetes 57: 3231–3238. 18776140 10.2337/db07-1690 PMC2584128

[21] Tuduri E, Marroqui L, Soriano S, Ropero AB, Batista TM, et al (2009) Inhibitory effects of leptin on pancreatic alpha-cell function. Diabetes 58: 1616–1624. 19401420 10.2337/db08-1787 PMC2699864

[22] Rajas F, Croset M, Zitoun C, Montano S, Mithieux G (2000) Induction of PEPCK gene expression in insulinopenia in rat small intestine. Diabetes 49: 1165–1168. 10909974 10.2337/diabetes.49.7.1165

[23] Ha J, Daniel S, Broyles SS, Kim KH (1994) Critical phosphorylation sites for acetyl-CoA carboxylase activity. J Biol Chem 269: 22162–22168. 7915280

[24] Mithieux G, Andreelli F, Magnan C (2009) Intestinal gluconeogenesis: key signal of central control of energy and glucose homeostasis. Curr Opin Clin Nutr Metab Care 12: 419–423. 19474723 10.1097/MCO.0b013e32832c4d6a

[25] van Schaftingen E, Gerin I (2002) The glucose-6-phosphatase system. Biochem J 362: 513–532. 11879177 10.1042/0264-6021:3620513 PMC1222414

[26] Hayek A, Woodside W (1979) Correlation between morphology and function in isolated islets of the Zucker rat. Diabetes 28: 565–569. 376379 10.2337/diab.28.6.565

[27] Himms-Hagen J, Harper ME (2001) Physiological role of UCP3 may be export of fatty acids from mitochondria when fatty acid oxidation predominates: an hypothesis. Exp Biol Med (Maywood) 226: 78–84. 11446442 10.1177/153537020122600204

[28] Vaughan RA, Garcia-Smith R, Gannon NP, Bisoffi M, Trujillo KA, et al.. (2013) Leucine treatment enhances oxidative capacity through complete carbohydrate oxidation and increased mitochondrial density in skeletal muscle cells. Amino Acids PMID:23812674 [Epub ahead of print] 10.1007/s00726-013-1538-5 23812674

[29] Bruckbauer A, Zemel MB, Thorpe T, Akula MR, Stuckey AC, et al (2012) Synergistic effects of leucine and resveratrol on insulin sensitivity and fat metabolism in adipocytes and mice. Nutr Metab (Lond) 9: 77. 22913271 10.1186/1743-7075-9-77 PMC3506499

[30] Newgard CB, An J, Bain JR, Muehlbauer MJ, Stevens RD, et al (2009) A branched-chain amino acid-related metabolic signature that differentiates obese and lean humans and contributes to insulin resistance. Cell Metab 9: 311–326. 19356713 10.1016/j.cmet.2009.02.002 PMC3640280

[31] Hardy OT, Czech MP, Corvera S (2012) What causes the insulin resistance underlying obesity? Curr Opin Endocrinol Diabetes Obes 19: 81–87. 22327367 10.1097/MED.0b013e3283514e13 PMC4038351

[32] Eller LK, Saha DC, Shearer J, Reimer RA (2013) Dietary leucine improves whole-body insulin sensitivity independent of body fat in diet-induced obese Sprague-Dawley rats. J Nutr Biochem 24: 1285–94. 23332601 10.1016/j.jnutbio.2012.10.004

[33] Qin LQ, Xun P, Bujnowski D, Daviglus ML, Van Horn L, et al (2011) Higher Branched-Chain Amino Acid Intake Is Associated with a Lower Prevalence of Being Overweight or Obese in Middle-Aged East Asian and Western Adults. J Nutr 141: 249–54. 21169225 10.3945/jn.110.128520 PMC3021443

