# Supplementary material for: Leucine Supplementation Protects from Insulin Resistance by Regulating Adiposity Levels
Source: PLoS One. 2013 Sep 25;8(9):e74705. doi: 10.1371/journal.pone.0074705 (PMC3783457; doi:10.1371/journal.pone.0074705)
Supplement: Table S1 — Mouse qPCR primer sequences. (DOC) [file pone.0074705.s001.doc]

**Table S1.** Mouse qPCR primer sequences.

| **Gene** | **GenBank ID** | **Forward sequence (5’-3’)** | **Reverse sequence (5’-3’)** |
| --- | --- | --- | --- |
| ACC-1 | NM_133360 | ATTGACCCAGACTGGCTTGAA | GTGTGAAGGCTGCTTTGTGAAC |
| Actg1 | NM_009609 | CAGCCTGGTGGATCTCTGTGA | CCTGGAAGTCTGCTGTTGGTTAA |
| Adrβ1 | NM_007419 | TTCTCCTAGAGGGCAAACCTTGT | CAGAGTGAGGTAGAGGACCCACA |
| Adrβ2 | NM_007420 | CTGTGCCTTCGCAGGTCTTC | TCCGTTCTGCCGTTGCTATT |
| Adrβ3 | NM_013462 | CGACATGTTCCTCCACAAATCA | TGGATTCCTGCTCTCAAACTAACC |
| CPT-1 | NM_009948 | AGCCTCTCCACCAGCCAGAT | CAGATGATTGGGATACTGTTTTGG |
| Cox-III | NC_005089 | TGACCCACCAAACTCATGCATA | GGGCTGAAAAGGCTCCAGTTA |
| Cox-IV | NM_053091 | ACCATCGCTCCAACGAATG | CACCAGAGCCGTGAATCCA |
| FAS | NM_007988 | GTTGGAAGTCAGCTATGAAGCAATT | AGACGCCAGTGTTCGTTCCT |
| GAPDH | NM_008084 | TCAAGAAGGTGGTGAAGCAG | TGGGAGTTGCTGTTGAAGTC |
| NRF-1 | NM_010938 | CACAGGACCTCTGCGCAAA | TGCCTGGGAAAAGTCCTCAC |
| Ppia | NM_008907 | CAAATGCTGGACCAAACACAA | GCCATCCAGCCATTCAGTCT |
| TFAM | NM_009360 | TCGGAGACCTACCTCAGATTAAGTG | AGAGGAGGGAGGAAGCCTGAT |
| UCP-1 | NM_009463 | AAAAAGAGCTGATGAAGTCCAGACA | TCAGTATCTCTTCCTCCAAGTTGCT |
| UCP-3 | NM_009464 | ACCCACATGGTGGAAGGACA | TGTATAGGGCGCTCAAATGGA |
| Ywhaz | NM_011740 | CTTGTGAGGCTGTGACACAAACA | CAAGAGTGTGCACGCAGACA |

ACC-1: Acetyl-CoA carboxylase; Actg1: actin gamma, cytoplasmic 1; Adrβ1, Adrβ2, Adrβ3: β1-, β2-, β3-adrenergic receptor; CPT-1: Carnitine palmitoyltransferase-1; Cox-III: Cytochrome c oxidase subunit 3; Cox-IV: Cytochrome c oxidase subunit 4; FAS: fatty acid synthase; GAPDH: Glyceraldehyde-3-phosphate dehydrogenase; NRF-1: nuclear respiratory factor; Ppia: peptidylprolyl isomerase A; TFAM: Transcription factor A, mitochondrial; UCP-1, UCP-3: uncoupling protein-1, - 3; Ywhaz: tyrosine 3 mono oxygenase tryptophan 5 mono oxygenase activation protein, zeta polypeptide.

N.B. GAPDH and Ppia were used as reference genes for the brown adipose tissue; Actg1 and Ywhaz were used as reference genes for the white adipose tissue.
